# Approaching Higher Dimension Imaging Data Using Cluster-Based Hierarchical Modeling in Patients with Heart Failure Preserved Ejection Fraction

**DOI:** 10.1038/s41598-019-46873-7

**Published:** 2019-07-18

**Authors:** Yukari Kobayashi, Maxime Tremblay-Gravel, Kalyani A. Boralkar, Xiao Li, Tomoko Nishi, Myriam Amsallem, Kegan J. Moneghetti, Sara Bouajila, Mona Selej, Mehmet O. Ozen, Utkan Demirci, Euan Ashley, Matthew Wheeler, Kirk U. Knowlton, Tatiana Kouznetsova, Francois Haddad

**Affiliations:** 1 Division of Cardiovascular Medicine, Stanford University School of Medicine, Stanford, CA United States; 2 Stanford Cardiovascular Institute, Stanford, CA United States; 3 Medical Director, Franchise, Medical Affairs Strategy, Actelion Pharmaceuticals US, Inc South, San Francisco, California, United States; 4 Bio-Acoustic -MEMS in Medicine (BAMM) Laboratories, Canary Center at Stanford for Cancer Early Detection, Department of Radiology, Stanford University School of Medicine, Palo Alto, CA United States; 5 Intermountain Medical Center Intermountain Heart Institute, Salt Lake City, UT United States; 6 Research Unit Hypertension and Cardiovascular Epidemiology, Department of Cardiovascular Sciences, Leuven, Belgium

**Keywords:** Cardiovascular biology, Outcomes research

## Abstract

Heart failure with preserved ejection fraction (HFpEF) is a major cause of morbidity and mortality, accounting for the majority of heart failure (HF) hospitalization. To identify the most complementary predictors of mortality among clinical, laboratory and echocardiographic data, we used cluster based hierarchical modeling. Using Stanford Translational Research Database, we identified patients hospitalized with HFpEF between 2005 and 2016 in whom echocardiogram and NT-proBNP were both available at the time of admission. Comprehensive echocardiographic assessment including left ventricular longitudinal strain (LVLS), right ventricular function and right ventricular systolic pressure (RVSP) was performed. The outcome was defined as all-cause mortality. Among patients identified, 186 patients with complete echocardiographic assessment were included in the analysis. The cohort included 58% female, with a mean age of 78.7 ± 13.5 years, LVLS of −13.3 ± 2.5%, an estimated RVSP of 38 ± 13 mmHg. Unsupervised cluster analyses identified six clusters including ventricular systolic-function cluster, diastolic-hemodynamic cluster, end-organ function cluster, vital-sign cluster, complete blood count and sodium clusters. Using a stepwise hierarchical selection from each cluster, we identified NT-proBNP (standard hazard ratio [95%CI] = 1.56 [1.17–2.08]) and RVSP (1.37 [1.09–1.78]) as independent correlates of outcome. When adding these parameters to the well validated Get with the Guideline Heart Failure risk score, the Chi-square was significantly improved (p = 0.01). In conclusion, NT-proBNP and RVSP were independently predictive in HFpEF among clinical, imaging, and biomarker parameters. Cluster-based hierarchical modeling may help identify the complementally predictive parameters in small cohorts with higher dimensional clinical data.

## Introduction

Heart failure (HF) with preserved ejection fraction (HFpEF) is a major cause of morbidity and mortality in the aging population, accounting for more than half of HF hospitalization^1–4^. Patients with HFpEF have a high prevalence of comorbidities including systemic hypertension, diabetes mellitus, chronic kidney disease, atrial fibrillation as well as sleep-disordered breathing.

The high mortality rate associated with HFpEF has driven several investigators to focus on identifying the best clinical and imaging predictors of outcome. Recent studies demonstrated the prognostic value of RV function^5,6^ and especially, Lam *et al*. showed that elevated right ventricular systolic pressure (RVSP) had a strong prognostic value among echocardiographic predictors^7^. There has also been a recent interest in left ventricular (LV) deformation imaging in HFpEF and most studies demonstrated that a significant percentage of patients with HFpEF have altered LV longitudinal strain (LVLS). LVLS was reported to be one of the predictors of prognosis^8–10^, however, the independent prognostic value of LVLS in HFpEF remains controversial^11^. In parallel, several validated scores predicting in-hospital mortality have been developed. Among these scores, the Get With The Guidelines-Heart Failure (GWTG-HF) risk score was shown to successfully predict in-hospital mortality^12,13^ as well as early post-discharge mortality risk^14^ based on clinical and biochemical variables. N-terminal pro-B-type natriuretic peptide (NT-proBNP) levels have also shown to be incremental to the GWTG-HF risk score to predict in-hospital mortality^12^ or mortality after discharge^15^.

To date few studies have evaluated as to which extent echocardiography is complementary to clinical and laboratory parameters or validated risk scores in patients with HFpEF. In this study, we sought to determine this complementarity of these parameters, first by using unsupervised cluster analysis and then hierarchical Cox regression modeling based on the identified clusters. We finally sought to evaluate whether the emerging parameters are additionally predictive to the GWTG-HF risk score.

## Methods

### Study design and patient population

Using the Stanford Translational Research Integrated Database Environment (STRIDE)^16^, we identified patients hospitalized for HFpEF (ICD-9 code 428.3) between January 2005 and December 2016 who had a transthoracic echocardiography and the data of NT-proBNP at the time of their hospitalization. STRIDE contains clinical information of pediatric and adult patients cared for at Stanford Health Care and Stanford Children’s Health including patients encounters with transcriptions of all inpatient and outpatient clinical notes, pathology and radiology reports, medication lists, lab results, and vitals data. This data source was accepted under approved Institutional Review Board protocols. Each chart was carefully reviewed by two physicians trained in data extraction (FH and KB) to ensure the diagnosis of acute HF according to the Framingham criteria^17^ and preserved LV systolic function defined as LVEF >50% was confirmed by echocardiographic report obtained near admission. Patients were excluded if they had a diagnosis of hypertrophic cardiomyopathy, pulmonary arterial hypertension, heart transplantation, pericardial disease, congenital heart disease, severe valvular heart disease or prior cardiac surgery. Patients with end-stage renal disease on dialysis, cirrhosis or active malignancy were excluded because of their influence on life expectancy. Patients were also excluded in the analysis if their LVLS, left atrial volume (LAV), LA strain, right ventricular free-wall longitudinal strain (RVLS), right atrial (RA) strain or RVSP was not measurable. We also randomly selected 50 age- and sex-matched controls from Stanford healthy aging cardiovascular institute database for purposes of comparison of specific echocardiographic parameters. This study was approved by the Stanford Institutional Review Board with all protocols conducted in accordance with relevant guidelines and regulations. Informed consent was obtained from all patients enrolled.

### Clinical and laboratory data

For each patient, we collected demographic, vital signs on admission, complete blood count (CBC), comprehensive metabolic panel and NT-proBNP values. NT-proBNP concentrations were obtained using Roche Biochemistry analyzer (Roche diagnostics, Mannheim, Germany). Other laboratory data at the time of admission included blood urea nitrogen (BUN), sodium, potassium concentration, white blood cell count, hemoglobin concentration, platelets count, red cell distribution width (RDW), and mean corpuscular volume (MCV). We also calculated the GWTG-HF risk score from a point-score system using age, systolic blood pressure, heart rate, black race and chronic obstructive pulmonary disease^13^.

### Echocardiography

Echocardiography was performed using commercially available echocardiographic systems (Sonos 7500, iE33, and EPIQ 7 C; Philips Medical Imaging, Eindhoven, the Netherlands), according to the American Society of Echocardiography guideline recommendations^18^. Image analyses were performed on Xcelera workstation by trained cardiologists from the Biomarker and Imaging Core laboratory at Stanford Cardiovascular Institute (YK, MTG). Standard echocardiographic views were obtained in two-dimensional (2D) and color tissue Doppler modes. LV end-diastolic volume, end-systolic volume and LV ejection fraction (EF) were calculated using Simpson’s method. Transmitral pulsed-wave Doppler velocity and tissue Doppler velocity of the lateral mitral annulus were obtained from apical 4-chamber view. LVLS was obtained using Lagrangian strain by manual tracing from the apical 4-chamber view. LA volumes were obtained using biplane area-length method and LA strain was calculated using Lagrangian strain. RV function was quantified using tricuspid annular plane systolic excursion (TAPSE), fractional area change (RVFAC) and RVLS using Lagrangian strain in a similar fashion. Right atrial pressure (RAP) was estimated as 3 mmHg if the inferior vena cava (IVC) diameter ≤2.1 cm and collapsed >50%, 15 mmHg if the IVC diameter >2.1 cm and collapsed <50%, and 8 mmHg in scenarios in which IVC diameter is enlarged or collapse index is sub-optimal otherwise. RVSP was estimated from the sum of the tricuspid regurgitation (TR) maximal velocity using the modified Bernoulli equation and estimated RAP. All strains were obtained using Lagrangian strain by tracing the myocardium manually and calculated in the following formula: 100 × (L_1_-L_0_)/L_0_^19–23^. LVLS was obtained with apical 4-chamber view, endo/epicardial circumferential strains with the parasternal short-axis view, RVLS with RV focused apical 4-chamber view in end-diastole (L_0_) and end-systole (L_1_). LA strain and RA strain were obtained with apical 4-chamber view at the time of maximum volume (L_0_) and minimum volume (L_1_). The intraclass correlation coefficient (ICC) of interobserver variability was 0.91 for LVLS, 0.91 for LA strain or 0.86 for RVLS and ICC of intraobserver variability was 0.99 for LVLS, 0.98 for LA strain, and 0.91 for RVLS in our Stanford Biomarker and Phenotypic Core Laboratory. In case of atrial fibrillation, the values from five cardiac cycles with similar RR interval were averaged. Area and volumes were indexed to body surface area.

### Outcome

The primary outcome was defined as all-cause mortality. The status was confirmed using chart review, care everywhere network (Epic Systems Corporation, Verona WI) or the United States Social Security Death Index to ascertain the vital status of each patient as of February 2014; and the time of the event was determined from whichever was later.

### Statistical analyses

Results are expressed as mean ± standard deviation for continuous variables or median and interquartile range when not normally distributed, or as the frequency and percentage for categorical variables. To delineate their relationship between parameters, we used unsupervised cluster analysis. Gephi was used for network visualization. The nodes are colored based on the modules. The thickness of the edges reflects the R-squared between two nodes. This unsupervised cluster analysis was used to guide the stepwise supervised analysis. Univariable Cox regression analysis was firstly performed to evaluate the association with the outcome for each parameter, then stepwise Cox regression analysis was performed to identify parameters that emerge in each cluster. The parameters retained in the previous model in each cluster were used in the final analysis. Hazard ratios and 95% confidence intervals were standardized by each standard deviation to compare the strength of association with outcome between parameters. LASSO analysis was also performed using the categorical end-point at 3 years to assess whether a different method yields similar findings^24^ (glmnet version 2.0–5). The predictive value of the model was assessed by the median of the AUROCs (area under the receiver operating characteristic) calculated in the test sets. Finally, the complemental value of these parameters to the GWTG-HF risk score was evaluated. P values < 0.05 were considered statistically significant. Analyses were performed using SPSS version 21 (SPSS Inc, Chicago, Illinois) and glmnet R package (glmnet version 2.0–5). (Full explanation of cluster analysis and LASSO analysis are shown in Supplemental Material).

## Results

**Figure 1:**
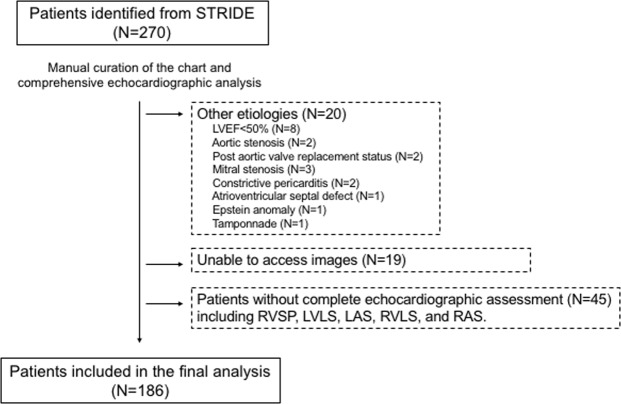
Flow chart of patients in the study. Patients were identified using Stanford Translational Research Integrated Database Environment (STRIDE). Manual curation of the chart was performed to confirm a diagnosis of heart failure for hospitalization. Echocardiographic images of each patient were reviewed to exclude other etiologies and comprehensive echocardiographic assessment was performed. If either one of the echocardiographic parameters of LVLS, LAV, LAS, RVLS, RAS or RVSP was not measurable due to the poor 2D image quality or the quality of tricuspid regurgitation signal. LAV, left atrial volume; LAS, left atrial strain; LVLS, left ventricular longitudinal strain; RAS, right atrial strain; RVLS, right ventricular longitudinal strain; RVSP, right ventricular systolic pressure.

From the STRIDE database, we identified 270 patients with a HFpEF diagnosis whose echocardiographic assessment and NT-proBNP were available at the time of admission. Of these patients, 39 patients were excluded because of the other etiologies (n = 20) or unable to access to the images (n = 19). Furthermore, for the study purpose of comprehensive echocardiographic assessment, 45 patients were excluded in the analysis because either one of RVSP, LVLS, LA strain, RVLS, or RA strain was not obtained due to the quality of tricuspid regurgitation signal or poor 2D images (Fig. 1). Finally, a total of 186 patients were included in the study. When comparing the patients in whom comprehensive imaging was available from the original cohort, the patients included in the analysis did not differ from the original cohort with regards to age (79 ± 13 vs. 78 ± 15, p = 0.42), sex (58% vs. 60% female, p = 0.56), comorbidity of diabetes mellitus (34% vs. 36%, p = 0.62), a history of coronary artery disease (60% vs. 61%, p = 0.92), the presence of atrial fibrillation/flutter (31% vs. 29%, p = 0.75) or overall mortality (32% vs. 33%, p = 0.92). Moreover, there was no significant difference in the GWTG-HF risk score (41.3 ± 7.3 vs. 41.8 ± 7.7, p = 0.55).

**Figure 2:**
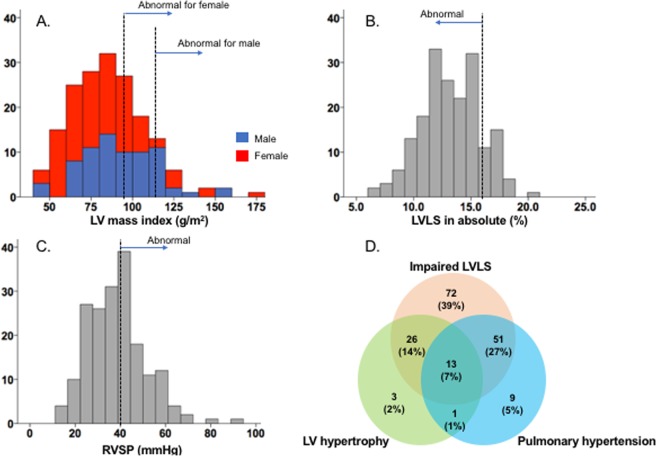
The prevalence of cardiac impairment in patients with HFPEF. The distribution of LV mass index (**A**), LVLS (**B**) and RVSP (**C**). The panel D presents the venn diagram demonstrating the overlap between LV hypertrophy (threshold of LV mass index 115 g/m^2^ for male and 95 g/m^2^ for female), impaired LVLS (threshold of LVLS −16%), and pulmonary hypertension (threshold RVSP of 40 mmHg) features. LVLS, left ventricular longitudinal strain; RVSP, right ventricular systolic pressure.

Table 1 shows the baseline characteristics of patients with HFpEF and controls. The mean age of patients with HFpEF was 78.7 ± 13.5 years with a majority of female patients (58%). Of 186 patients, 58 patients (31%) presented with atrial fibrillation or flutter when echocardiographic assessment was performed. Table 2 shows the echocardiographic assessment in patients with HFpEF. While all patients presented LVEF > 50%, only 24 patients (13%) presented with absolute LVLS > 16%. We used the cut-off value in LVLS as 16% in absolute value, based on previous literature where normal LVGLS ranged from 15.9 to 22.1% in absolute value^9,25^. LV hypertrophy defined as LVMI > 95 g/m^2^ for women and LVMI > 115 g/m^2^ for men was observed in 43 patients (23%) and pulmonary hypertension defined by RVSP ≥ 40 mmHg was observed in 75 patients (40%) (Fig. 2A–C). The comparison of these echocardiographic parameters between the patients with HFpEF and age- and sex-matched controls is shown in Supplemental Fig. 1. Figure 2D presents the Venn diagram demonstrating the overlap between the number of patients with LV hypertrophy, impaired LVLS, and pulmonary hypertension. These features were selected because of the importance of RVSP on outcome^7^ as well as the key characteristics of LV hypertrophy and decreased LVLS in patients with HFpEF. RV dysfunction based on TAPSE < 1.6 cm, RVFAC < 35% or absolute RVLS < 20% was present in 51 (27%), 45 (24%), or 47 patients (25%), respectively. We used the cut-off value in RVLS as 20% in absolute value, based on 95% lower limit of controls reported^26^.

**Table 1:** Clinical characteristics.

Parameters	Controls N = 50	HFpEF N = 186	P value
Age (years)	77.6 ± 6.4	78.7 ± 13.5	0.57
Male, n (%)	24 (48)	80 (43)	0.42
BSA (m^2^)	1.76 ± 0.32	1.88 ± 0.31	0.01
BMI (kg/m^2^)	23.3 ± 4.2	28.3 ± 7.7	<0.001
Heart rate (bpm)	58 ± 19	80 ± 18	<0.001
Systolic blood pressure (mmHg)	123 ± 18	135 ± 25	<0.001
Diastolic blood pressure (mmHg)	72 ± 11	68 ± 17	0.07
Atrial fibrillation/flutter, n (%)	0	58 (31)	<0.001
Hypertension, n (%)	14 (28)	186 (100)	<0.001
Diabetes mellitus, n (%)	0	62 (33)	<0.001
History of coronary artery disease, n (%)	0	112 (60)	<0.001
**Medication**			
Beta blocker, n (%)	1 (2)	108 (58)	<0.001
ACE-I/ARB, n (%)	10 (20)	73 (39)	0.02
Calcium channel blocker, n (%)	4 (8)	51 (27)	0.004
Diuretics, n (%)	6 (12)	117 (63)	<0.001
Spironolactone, n (%)	0	9 (5)	0.21
NT-proBNP (pg/dl)	N.A.	2151 (1075–4752)	N.A.

ACE-I; angiotensin converting enzyme inhibitor, ARB; angiotensin II receptor blocker, BMI; body mass index, BSA; body surface area.

**Table 2:** Echocardiographic measurements.

Parameters	N = 186
Interventricular septal thickness (cm)	1.1 ± 0.2
Posterior wall thickness (cm)	1.0 ± 0.2
LV internal diameter (cm)	4.5 ± 0.7
Relative wall thickness	0.47 ± 0.11
LV mass index (g/m^2^)	86.1 ± 23.3
LVEF (%)	62 ± 7
LVLS (%)	−13.2 ± 2.6
Epicardial circumferential strain (%)	−9.2 ± 2.9
Endocardial circumferential strain (%)	−31.3 ± 8.8
Lateral e’ (cm/s)	8.3 ± 2.9
Lateral E/e’	11.9 ± 5.2
Maximal LA volume index (ml/m^2^)	37.2 ± 16.0
LA emptying fraction (%)	40.4 ± 13.0
LA strain (%)	−15.8 ± 6.0
TAPSE (mm)	19.8 ± 6.0
RVFAC (%)	38.1 ± 6.6
RVLS (%)	−23.0 ± 4.9
RA emptying fraction (%)	39.9 ± 13.9
RA strain (%)	−19.4 ± 7.2
Maximal RA volume index (ml/m^2^)	32.1 ± 19.3
Maximal RA area index (cm^2^/m^2^)	10.6 ± 3.9
Estimated RVSP (mmHg)	38.5 ± 12.8

LA; left atrial, LVEF; left ventricular ejection fraction, LVLS; left ventricular longitudinal strain, NT-proBNP; N-terminal pro B-type natriuretic peptide, RA; right atrial, RVFAC; right ventricular fractional area change, RVSP; right ventricular systolic pressure.

### Unsupervised: Clustering and network analysis

**Figure 3:**
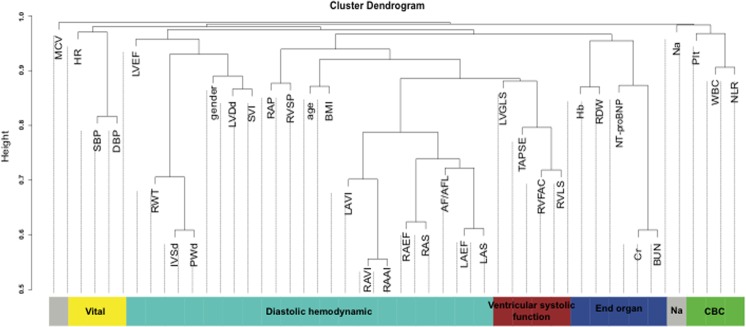
Cluster Dendrogram of clinical, laboratory and echocardiographic echocardiographic features. Dynamic tree cut algorithm detected six clusters of closely associated features shown in blue, brown, green, red, yellow, and turquoise. AF; atrial fibrillation, AFL; atrial flutter, BMI; body mass index, BUN; blood urea nitrogen, Cr; creatinine, DBP; diastolic blood pressure, eGFR; estimate glomerular filtration rate, Hb; hemoglobin, HR; heart rate, IVSd; diastolic interventricular septum, LAEF; left atrial emptying fraction, LAS; left atrial strain, LAVI; left atrial volume index, LVDd; diastolic left ventricular dimension, LVEF; left ventricular ejection fraction, LVLS; left ventricular longitudinal strain, LVMI; left ventricular mass index, MCV; mean corpuscular volume, Na; sodium, NLR; neutrophil-to-lymphocyte ratio, NT-proBNP; N-terminal pro B-type natriuretic peptide, Plt; platelet, PWd; diastolic posterior wall, RAAI; right atrial area index, RAEF; right atrial emptying fraction, RAP; right atrial pressure, RAS; right atrial strain, RAVI; right atrial volume index, RDW; red cell distribution width, RVFAC; right ventricular fractional area change, RVLS; right ventricular longitudinal strain, RVSP; right ventricular systolic pressure, RWT; relative wall thickness, SBP; systolic blood pressure, SVI; stroke volume index, TAPSE; tricuspid annular plane systolic excursion, WBC; white blood cell.

As shown in the cluster dendrogram of Fig. 3, we identified six clusters, each of which highlights parameters that are more closely associated with each other. These include the end-organ function cluster (blue) including NT-proBNP, creatinine, blood urea nitrogen, RDW and Hb; the ventricular systolic function cluster (red) including LVLS and RV functional parameters; the diastolic hemodynamic cluster (turquoise) including RVSP, right atrial pressure, and atrial size and strain metrics; the vital sign cluster; the CBC based cluster and the sodium based cluster.

**Figure 4:**
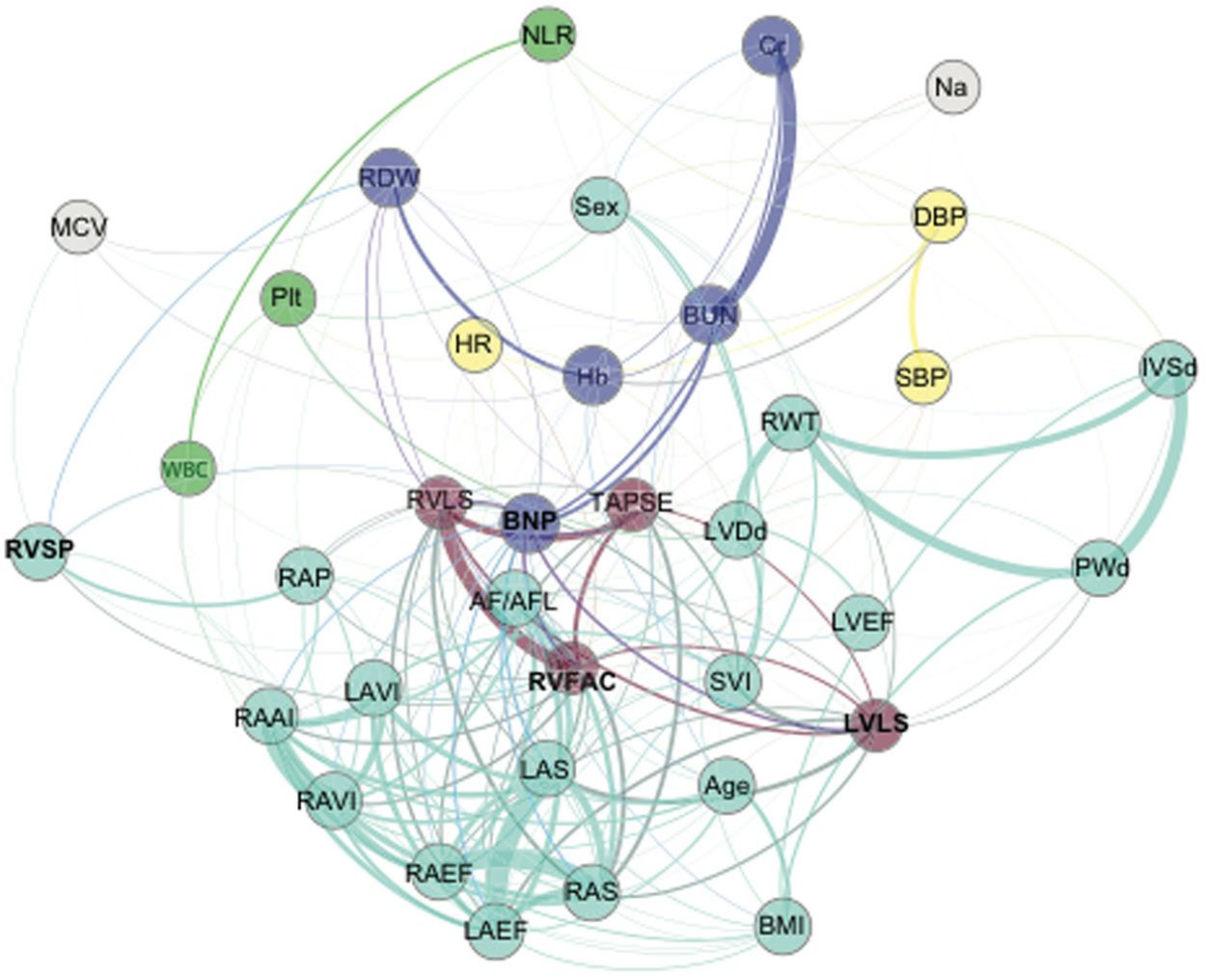
Network analysis of different clinical, laboratory and echocardiographic features. The nodes are colored based on the clusters in Fig. 3. The thickness of the edges reflects the topological overlap between two nodes. Abbreviation; same as Fig. 3.

Figure 4 shows the correlation and connection network between those parameters. NT-proBNP was centered being strongly connected with right ventricular function parameters while its connection with RVSP or LVLS was weak.

### Supervised hierarchical outcome analysis: cox regression analysis and LASSO analysis

During a median (IQR) follow-up of 3.3 (1.1–5.7) years, 60 patients (32%) died, among whom 7 patients (3.8%) died during hospitalization. Mortality rates were 17% at 1-year, 25% at 3-years and 47% at 5-years.

Table 3 presents the cluster based hierarchical modeling; first on the univariate Cox regression analysis followed by multivariable intra and inter cluster analysis. Among the stronger independent factors in each cluster, we identified NT-proBNP (standardized hazard ratio (SHR) [95%CI] = 1.58 [1.20–2.09], p = 0.001) as an independent associate in the end-organ function cluster, RVFAC (1.38 [1.09–1.74], p = 0.008) as an independent associate in ventricular systolic cluster, and age (1.38 [1.00–1.88], p = 0.05), RA strain (1.30 [1.00–1.69], p = 0.049) and RVSP (1.44 [1.12–1.86], p = 0.005) as independent associates in diastolic hemodynamic cluster. The parameters in the other clusters were not significantly associated with the outcome in our cohort. The final multivariable analysis demonstrated that NT-proBNP (1.56 [1.17–2.08], p = 0.003) and RVSP (1.37 [1.09–1.73], p = 0.008) were independent associates with the outcome. The above results were further supported by Kaplan-Meier curve analysis which showed NT-proBNP (Log-rank p = 0.03) and RVSP (Log-rank p = 0.02) differentiated the outcome according to their tertile (Supplemental Fig. 2). LASSO analysis also found NT-proBNP and RVSP as associates with outcome (AUROC = 0.67).

**Table 3:** Parameters are listed in descending order of standard hazard ratio within each cluster.

	SHR	95% CI	P	SHR	95% CI	P	SHR	95% CI	P
	Univariable			Multivariable in cluster			Overall multivariable		
**End-organ function cluster**									
Log NT-pro BNP per 1SD increase	1.58	1.20–2.09	0.001	1.58	1.20–2.09	0.001	1.56	1.17–2.08	0.003
BUN per 1SD increase	1.26	1.02–1.55	0.004						
Hb per 1SD increase	1.18	0.92–1.51	0.18						
RDW per 1SD increase	1.14	0.94–1.39	0.18						
Creatinine per 1SD decrease	1.04	0.82–1.34	0.74						
**Ventricular systolic cluster**									
RVFAC per 1SD decrease	1.38	1.09–1.74	0.008	1.38	1.09–1.74	0.008			
LVLS per 1SD worsening	1.34	1.05–1.73	0.02						
TAPSE per 1SD decrease	1.30	0.99–1.70	0.06						
RVLS per 1SD worsening	1.28	0.99–1.65	0.06						
**Diastolic hemodynamic cluster**									
Age per 1SD increase	1.47	1.07–2.01	0.02	1.38	1.00–1.88	0.050			
RAS per 1SD worsening	1.41	1.01–1.82	0.009	1.30	1.00–1.69	0.049			
RVSP per 1SD increase	1.41	1.12–1.76	0.003	1.44	1.12–1.86	0.005	1.37	1.09–1.73	0.008
LAS per 1SD worsening	1.38	1.05–1.81	0.02						
LAEF per 1SD worsening	1.37	1.05–1.77	0.02						
BMI per 1SD increase	1.34	0.99–1.80	0.05						

If the number of parameters in the cluster is more than 6, only 6 parameters with higher standardized hazard ratio were listed in the Table. The parameters in the other clusters were not listed because all of the parameters were not significantly associated with the outcome.

BMI; body mass index, LAS; left atrial strain, LAVI; left atrial volume index, LVLS; left ventricular longitudinal strain, NT-proBNP; N-terminal pro B-type natriuretic peptide, RAS; right atrial strain, RDW; red cell distribution width, RVFAC; right ventricular fractional area change, RVLS; right ventricular longitudinal strain, RVSP; right ventricular systolic pressure, SHR; standardized hazard ratio, TAPSE; tricuspid annular plane systolic excursion.

### Analyzing the value of right heart metrics-RVSP ratios

 Due to the association with outcome of RVSP and right heart metrics in our cohort and the recent reports of the importance of the relationship between RV contractile function and RVSP or its coupling to pulmonary circulation in heart failure^27,28^, we also tested whether TAPSE/RVSP ratio, RVFAC/RVSP ratio, RVLS/RVSP ratio or RAS/RVSP ratios would carry more predictive value when compared to RVSP alone. Cox regression analysis demonstrated that all of those ratios predicted outcome comparably or even better than RVSP alone (SHR [95%CI] = 1.57 [1.15–2.14] per 1 SD worsening, p = 0.004 for TAPSE/RVSP ratio, 1.66 [1.20–2.29] per 1 SD worsening, p = 0.002 for RVFAC/RVSP ratio, 1.57 [1.14–2.16] per 1 SD worsening, p < 0.001 for RVLS/RVSP ratio, 1.81 [1.30–2.53] per 1 SD worsening, p < 0.001 for RAS/RVSP ratio and 1.41 [1.12–1.76] per 1 SD worsening, p = 0.003 for RVSP alone).

### Complementary value to validated clinical score

**Figure 5:**
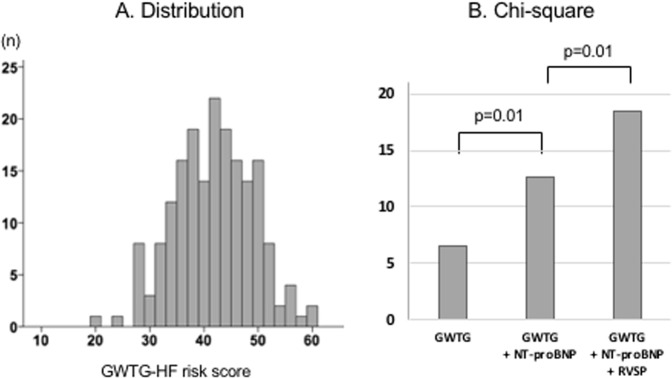
The GWTG-HF risk score and its complementarity to RVSP and NT-proBNP. (**A**) Distribution of GWTG-HF risk score. (**B**) Chi-square comparison between the models with the GWTG-HF risk score alone, adding NT-proBNP, and further adding RVSP.

The mean value of the GWTG-HF risk score was 41.3 ± 7.3 (Fig. 5A) and the score predicted long-term outcome in our cohort (SHR [95%CI] = 1.73 [1.36–2.22], p < 0.001). The chi-square in the models to predict outcome using the GWTG-HF risk score only, the GWTG-HF risk score and NT-proBNP, and the GWTG-HF risk score, NT-proBNP, and RVSP significantly improved adding these two parameters to the GWTG-HF risk score (Fig. 5B).

## Discussion

In this study, we used unsupervised cluster analysis method to guide hierarchical supervised outcome modeling in patients with acute HFpEF. This method allowed to identify the most predictive parameters among closely related metrics. NT-proBNP which was centrally connected among echocardiographic parameters and biomarkers was strongly prognostic for patients with HFpEF complementary with RVSP which captures hemodynamic severity.

HFpEF is a major cause of morbidity and mortality accounting for up to half patients with HF and the survival of patients with HFpEF is similar to that of patients with reduced ejection fraction^1,2^. Consistent with previous studies, patients in our study had a high prevalence of multimorbidity and the mortality rate was high, approaching 47% at 5-years.

With the emergence of higher dimensional imaging data in HF outcome analysis, understanding the complementarity may help better selected parameters for building multivariable modeling. This is a novel approach which is important especially for small cohorts where overfitting may be a major issue. Unsupervised cluster analysis is the task of grouping a set of parameters according to similarities and has been often used to display the genome-wide expression patterns. Since echocardiographic parameters relate with each other although the number is relatively small compared with the genes, this analysis may allow pattern classification that can then guide hierarchical modeling by analyzing the complementarity present both intra and inter clusters.

Our study identified the six clusters which capture key physiological domains in HFpEF. Among them, parameters in the end-organ function cluster, ventricular systolic function cluster, and diastolic hemodynamic cluster showed the relation with outcome. In the end-organ cluster, NT-proBNP and renal function emerged as the two strongest factors associated with outcome. Plasma levels of NT-proBNP are well-known biomarkers for neurohormonal activity in patients with HF and can be reliably used for diagnosis and the risk stratification in patients with HF, regardless of the LVEF^29,30^. Renal function has been also reported to be associated with adverse outcome in patients with acute and chronic heart failure^31,32^.

In the ventricular systolic function cluster, both LVLS and metrics of right heart function were associated with outcome. In patients with HFpEF, most patients presented with reduced LVLS, which has been controversial for predicting outcome^8–11,33,34^. In our study, LVLS was not retained in the multivariable model among the parameters in the ventricular systolic function cluster, probably because of its moderate correlation with RV function.

In the diastolic hemodynamic cluster, RVSP and RAS were independent predictors of outcome. RVSP has reported to be predictive in patients with HFpEF by Lam *et al*., who investigated 244 patients with HFpEF in their first population-based study^7^. They showed that patients with pulmonary artery systolic pressure (PASP) ≥ 48 mmHg had worse mortality than the patients with PASP < 48 mmHg for long-term follow-up. One of the interesting results in this cluster was that RAS was also strongly predictive for outcome. Given that the normal atrial function compensates to maintain ventricular filling with greater atrial compliance and atrial pump function, patients with reduced RA function are more likely to experience overt right heart failure, leading to worse outcome. This might be also supported by the results that RAS/RVSP ratio presented best standardized hazard ratio beyond RVSP alone among right heart function to RVSP ratios. Fewer researches have been performed how to assess RA function accurately by echocardiography, therefore, future studies are warranted to prove this finding.

While the method presented may be well suited to select the most predictive parameters, we may not validate previously identified predictors. As such, to the best of our knowledge, this study is the first to test whether RVSP and NT-proBNP would be incremental to the validated GWTG-HF risk score. This proved to improve the risk stratification for patients hospitalized with HFpEF beyond biomarker data alone, echocardiographic parameters alone, or clinical data alone. Since the GWTG-HF risk score does not include the information of those parameters, this study highlights the importance of combining hemodynamic severity and cardiac biomarker with the GWTG-HF risk score.

There are several limitations in this study. First, our sample size was relatively small and nearly 30% of patients had to be excluded because of incomplete data, as the study goal required comprehensive echocardiographic parameters. However, the baseline characteristics of patients excluded did not differ from the original cohort. This point is a challenge in usual clinical setting as imaging quality may be sub-optimal in the acute setting. Second, the RVSP was only estimated using the continuous Doppler measurements of the TR signal, without invasive confirmation. However, the reliability of this estimation has previously been reported when attention is given^35^ and each signal was reanalyzed with attention to signal quality. Third, we did not include E/e’ in this study as it was not systematically recorded in many of the patients whose echocardiography was performed before 2008. However, it was not significantly associated with the outcome in patients with E/e’ available in our study, therefore, we did not use E/e’ for further analysis. Furthermore, even in the patients with e’, it was acquired from only lateral annulus. More precise assessment will be needed in future studies. Fourth, we did not include cardiovascular death or rehospitalization as a secondary end-point since patients were followed at different institutions during the study period or we only know the vital status from the United States Social Security Death Index, leading to incomplete data collection. Finally, this is a single-center observation, therefore, further examination across multi-centers would be warranted to validate our present findings.

## Conclusion

Cluster-based hierarchical modeling may help understand higher dimensional data in small cohorts. This study identified the two most predictive factors in HFpEF among clinical, imaging, and biomarker parameters and demonstrates their potential incremental value to well validated GWTG-HF risk score.

## Supplementary information

Supplemental Materials

## Data Availability

The datasets generated during and/or analyzed during the current study are available from the corresponding author on reasonable request.
