# Supplementary material for: Approaching Higher Dimension Imaging Data Using Cluster-Based Hierarchical Modeling in Patients with Heart Failure Preserved Ejection Fraction
Source: Sci Rep. 2019 Jul 18;9:10431. doi: 10.1038/s41598-019-46873-7 (PMC6639369; doi:10.1038/s41598-019-46873-7)
Supplement: Supplementary file 1 — Supplemental Materials [file 41598_2019_46873_MOESM1_ESM.docx]

**Approaching Higher Dimension Imaging Data Using Cluster-Based Hierarchical Modeling in Patients with Heart Failure Preserved Ejection Fraction**

Yukari Kobayashi MD^1,2a^, Maxime Tremblay-Gravel MD MSc^1,2a^, Kalyani A Boralkar MD^1,2^, Xiao Li PhD^1^,  Tomoko Nishi MD^1,2^, Myriam Amsallem MD MSc^1,2^, Kegan J Moneghetti MBBS (hons)^1,2^, Sara Bouajila MD MSc^1,2^, Mona Selej, MD^3^,  Mehmet O. Ozen PhD^2,4^, Utkan Demirci PhD^2,4^, Euan Ashley MD^1,2^, Matthew Wheeler MD^1,2^, Kirk U Knowlton MD^5^, Tatiana Kouznetsova MD PhD^6^, Francois Haddad MD^1,2^

**Affiliations**

1. Division of Cardiovascular Medicine, Stanford University School of Medicine, Stanford, CA

2. Stanford Cardiovascular Institute, Stanford, CA

3 Medical Director, Franchise, Medical Affairs Strategy, Actelion Pharmaceuticals US, Inc

South San Francisco, California

4 Bio‐Acoustic ‐MEMS in Medicine (BAMM) Laboratories, Canary Center at Stanford for Cancer Early Detection, Department of Radiology, Stanford University School of Medicine, Palo Alto, CA, USA

5 Intermountain Medical Center Intermountain Heart Institute, Salt Lake City, UT

6 Research Unit Hypertension and Cardiovascular Epidemiology, Department of Cardiovascular Sciences, Leuven, Belgium

a. Both authors contributed equally to this study

Key words: Heart failure preserved ejection fraction, Echocardiography, Cluster analysis

**Running Title:** Cluster Based Hierarchical Modeling in HFpEF

**Address for correspondence:** Yukari Kobayashi, MD, Instructor of Medicine,  Stanford Cardiovascular Institute, 300 Pasteur Dr H2170, Stanford, CA 94305, Email: yukariko@stanford.edu

**-Supplemental methods**

Statistical analyses

Results are expressed as mean ± standard deviation for continuous variables or median and interquartile range when not normally distributed, or as the frequency and percentage for categorical variables. To delineate their relationship between parameters, we used unsupervised cluster analysis. Specifically, WGCNA R package (WGCNA version 1.63) was applied and the following steps were performed for network construction and analysis: (1) an adjacency matrix was formed by calculating pairwise correlation (R-squared) of any two parameters;  (2) a topological overlap matrix was then calculated from the adjacent network and used to (3) build hierarchical clustering tree to identify modules using Dynamic Tree Cut algorithm. Gephi was used for network visualization. The nodes are colored based on the modules. The thickness of the edges reflects the R-squared between two nodes. For visualization purpose, edges of R-squared <0.01 were not shown. This unsupervised cluster analysis was used to guide the stepwise supervised analysis. Univariable Cox regression analysis was firstly performed to evaluate the association with the outcome for each parameter, then stepwise Cox regression analysis was performed to identify parameters that emerge in each cluster. The parameters retained in the previous model in each cluster were used in the final analysis. The proportional hazard assumption was tested using scaled Schoenfeld residuals to assess the non-significance of the correlation against time of variables included in the multivariable models. Hazard ratios and 95% confidence intervals were standardized by each standard deviation to compare the strength of association with outcome between parameters. LASSO analysis was also performed using the categorical end-point at 3 years to assess whether a different method yields similar findings. Then, LASSO-penalized logistic regression was performed using glmnet R package [[21]](https://paperpile.com/c/qonmPx/wFu6) (glmnet version 2.0-5) to classify the patients based on their outcome and identify a minimum feature set to best explain the classification. A nested cross-validation procedure (5X5X5) was applied to avoid overfitting. Specifically, we split the positive and negative sets into five equal size bins, trained the LASSO-regression on four of them and evaluated the model on the remaining one. To avoid potential bias on the split, the above procedure was repeated five times after randomizing both sets. In addition, we applied a five-fold cross-validation procedure in each training process to learn the hyper-parameter *lambda*. The predictive value of the model was assessed by the median of the AUROCs (area under the receiver operating characteristic) calculated in the test sets. Finally, the complemental value of these parameters to GWTG-HF risk score was evaluated. P values < 0.05 were considered statistically significant. Analyses were performed using SPSS version 21 (SPSS Inc, Chicago, Illinois) and glmnet R package (glmnet version 2.0-5).

**-Supplemental Figure**

**Distribution of echocardiographic parameters in controls and HFpEF population**

Patients with HFpEF presented with higher RWT (A), LVMI (B), lower absolute LVLS (C), and larger LAVI (D) compared to controls.

LAVI; left atrial volume index, LVLS; left ventricular longitudinal strain, LVMI; left ventricular mass index, RWT; relative wall thickness

**Kaplan-Meier curves analysis in NT-proBNP and RVSP according to their tertile**

NT-proBNP (Log-rank p=0.03) and RVSP (Log-rank p=0.02) differentiated the all-cause mortality according to the tertile.
